# 2020年以来高效色谱材料的开发及其在本草物质组中的应用

**DOI:** 10.3724/SP.J.1123.2025.08025

**Published:** 2026-08-08

**Authors:** Jing FENG, Donghai XIA, Chunying SONG, Yi QI, Gaowa JIN, Yanfang LIU, Xinmiao LIANG, Dongping YU, Zhimou GUO

**Affiliations:** 1. 中国科学院大连化学物理研究所，植物化学与天然药物全国重点实验室，辽宁 大连 116023; 1. State Key Laboratory of Phytochemistry and Natural Medicines，Dalian Institute of Chemical Physics，Chinese Academy of Sciences，Dalian 116023，China; 2. 中国科学院大学，北京 100049; 2. University of Chinese Academy of Sciences，Beijing 100049，China; 3. 赣江中药创新中心，江西 南昌 330000; 3. Ganjiang Chinese Medicine Innovation Center，Nanchang 330000，China

**Keywords:** 色谱分离材料, 本草物质组, 分离分析, 制备色谱, chromatographic separation materials, herbalome, separation and analysis, preparative chromatography

## Abstract

中药作为中华民族的文化瑰宝，在多种疾病治疗中发挥着重要作用。由于中药化学成分极其复杂，全面解析物质基础和阐明药效机制是中药现代化研究面临的重要挑战。为了深入解析中药的物质基础，阐述中药的多组分多靶点整体调节机制，梁鑫淼等提出了本草物质组学的概念，涵盖物质基础与功能机制两大方向，包括中药化合物的定性与定量分析、活性成分分离以及其生物效应与作用机制的深入研究。在本草物质组研究中，色谱分离分析和制备纯化技术是解析中药物质基础的有效手段，而色谱分离材料的发展是提升中药复杂体系分离能力的关键驱动因素。本文系统综述了2020年以来新型高效色谱分离材料的开发及其在本草物质组研究中的应用，重点围绕反相液相色谱、亲水作用液相色谱和超临界流体色谱3种色谱模式，阐述了各类新型材料的结构和性能特点，以及在生物碱、黄酮、皂苷和萜类等关键活性成分分离分析与制备纯化中的应用。最后，文章对未来发展方向进行了展望，指出未来色谱材料的发展应聚焦于提升分离选择性与正交性、提高分离效率与制备载量、开发通用型固定相，以更好地支撑本草物质组研究的深入发展。本文旨在为中药复杂体系的物质基础解析提供材料基础和技术参考。

## Abstract (en)

Traditional Chinese medicine （TCM）， a treasured cultural heritage of the Chinese nation， plays a significant role in treating various diseases. However， the extreme complexity of its chemical components makes the comprehensive elucidation of the material basis and pharmacological mechanisms a major challenge in modern TCM research. To deeply investigate the material basis of TCM and interpret its holistic regulation mechanism involving multiple components and targets， Liang Xinmiao and his colleagues proposed the concept of herbal materiomics， which focuses on two main research directions： the material basis （including qualitative/quantitative analysis and isolation of active compounds） and the functional mechanisms （in-depth study of biological effects and modes of action）. In herbalome research， chromatographic analysis and preparative purification are powerful tools for deciphering the composition of complex TCM systems， with the development of novel chromatographic separation materials being a key driver of progress. This review systematically summarizes recent advances in the development of novel high performance chromatographic separation materials since 2020 and their applications in herbalome research. It focuses on three chromatographic modes： reversed-phase liquid chromatography （RPLC）， hydrophilic interaction liquid chromatography （HILIC）， and supercritical fluid chromatography （SFC）. The structural and performance characteristics of these novel materials are highlighted， along with their applications in the analysis and preparative purification of key active components such as alkaloids， flavonoids， saponins and terpenoids. In RPLC， the separation selectivity and retention capacity of the stationary phases for compounds such as alkaloids and flavonoids have been effectively enhanced through two primary strategies： first， by incorporating polar groups such as fluorine atoms， amides， carboxyl groups into the traditional C18/C8/phenyl skeletons； second， by utilizing natural product derivatives like cardanol and rosin as functional ligands. Furthermore， this approach has led to the creation of novel materials possessing orthogonal separation characteristics. In HILIC， various functionalized stationary phases have been developed， including *β*-cyclodextrin， nitrogen-containing heterocycles， core-shell structured amides， and surface-polymerized phases. These phases significantly improve the retention and separation efficiency of strongly polar compounds （e.g.， saponins， oligosaccharides） by providing abundant hydrogen bonding， electrostatic， and *π-π* interactions. In SFC， the development of novel stationary phases extends from modified functional skeletons （e.g.， phenyl） to zwitterionic and nitrogen-containing heterocyclic hydrophilic stationary phases. Additionally， mixed-mode stationary phases have been developed to effectively ameliorate the peak tailing problems associated with basic compounds. Concurrently， the emergence of small-particle-size ordered mesoporous silica materials enables ultra-high column efficiency and rapid separation of components such as tanshinones. This review further illustrates the successful application of these novel materials in both high-performance analytical and preparative chromatography， using four important classes of TCM active ingredients—alkaloids， flavonoids， saponins， and terpenoids—as examples. These novel materials afford significant chromatographic improvements—notably in separation selectivity， peak symmetry， analysis speed， and the resolution of previously difficult-to-separate isomers. Critically， the integration of these advanced materials has facilitated the construction of highly orthogonal multidimensional chromatographic systems （e.g.， RPLC×RPLC， HILIC×RPLC）， thereby providing the capacity for in-depth profiling and identification of dozens to hundreds of components from complex TCM extracts. In the field of preparative chromatography， these materials enable efficient and large-scale purification of target monomers， including trace active ingredients， thereby significantly enriching compound libraries for herbalome research. This includes the implementation of sophisticated multi-dimensional and multi-mode preparative strategies， which have facilitated the discovery of novel compounds and unprecedented skeletons. Finally， the article concludes by outlining future development directions of chromatographic materials， emphasizing the need to enhance separation selectivity and orthogonality， improve separation efficiency and preparative loading capacity， and develop versatile stationary phases. These advancements are crucial to better support the in-depth development of herbalome research. This review aims to provide a material foundation and technical reference for resolving the material basis of complex TCM systems.

中医药作为中华民族的文化瑰宝，具有丰富的药用价值和长期广泛的临床实践经验^［1］^。国家高度重视中医药发展，致力于推动中医药的现代化和国际化^［2］^。然而，由于中药化学成分的复杂性和药效物质基础的不确定性，中药现代化仍然面临着严峻的挑战^［3，4］^。因此，需要采用科学的理论和方法，解析中药的物质组成和结构，研究其作用机理，推动其疗效得到充分发挥和认可^［5］^。

中药物质组成结构极其复杂，主要体现在化合物种类多、极性分布广、含量差异大和分子结构相近等方面。因此，解析中药的物质组成结构一直是中药研究的一项巨大挑战，这也是制约中药深入研究的主要原因之一。为了深入研究中药的物质基础，阐述中药的多组分多靶点整体调节机制，梁鑫淼等^［6］^提出本草物质组学概念，旨在运用现代科学技术与方法，从基础分子的作用机制回答中医药治疗疾病的科学性，为中药现代化发展提供理论基础。本草物质组的研究聚焦于物质组成和功能两个层面：一是物质组成基础，对中药各种化合物定性定量分析，解析中药的物质组成和结构，分离纯化得到潜在活性化合物；二是功能机制研究，对得到的潜在活性化合物进行深入研究，探讨其在机体上作用的调节机制^［7］^。

在本草物质组研究中，色谱技术是解析中药复杂体系组成结构的有效手段。作为现代分离科学的重要分支，色谱技术基于物质在固定相和流动相间分配平衡的差异，实现对中药复杂体系中各类化学成分的高效分离。色谱分离材料的发展是提升复杂体系分离能力的关键驱动因素。针对中药化合物种类多、极性分布广、含量差异大和分子结构相近等特点，当前色谱分离材料聚焦于以下4个方向：改善色谱峰形，提高分离选择性，加快分析速度以及扩大化合物覆盖范围。近年来，这些新型分离材料在中药的分离分析与制备纯化研究中取得了显著进展。本文系统综述了2020年至今高效色谱分离材料的进展及其在本草物质组研究中的应用情况，包括高效色谱分离材料的发展、分离材料在中药成分的分离分析和制备纯化中的应用（图1）。

**图1:**
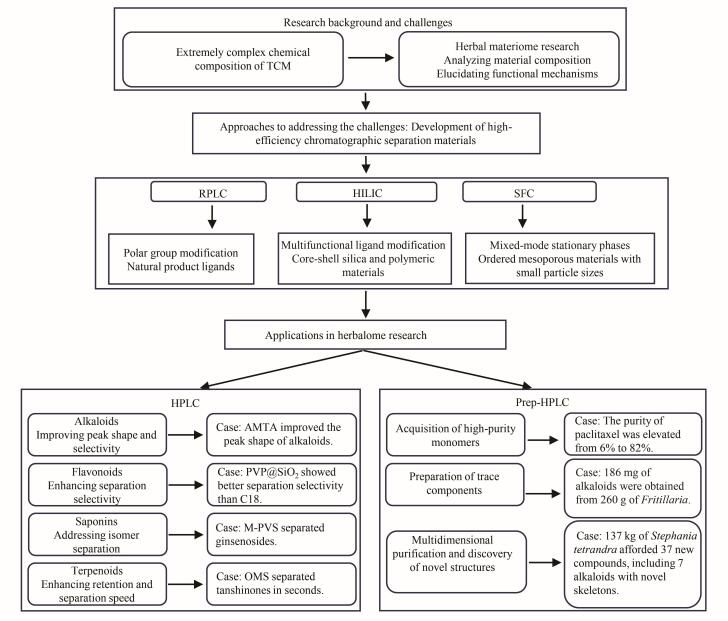
全文概要数据图

## 1 高效色谱分离材料的发展

中药物质组成结构极其复杂，主要体现在以下4个方面：1）化合物种类繁多，包含成百上千种不同结构类型的化学成分；2）极性分布范围广，从极性强的糖苷类化合物到极性弱的萜类化合物均有；3）化合物的含量跨度显著，差异可达多个数量级，从常量、微量和痕量都有分布；4）存在大量结构相似的同分异构体和同系物，其差异往往仅体现在单个官能团或立体构型上。中药的物质组成结构高度复杂，为其分离鉴定带来了巨大挑战。然而，这种固有的化学多样性，却为发现具有生物活性的药物分子提供了独特的资源体系。

为深入解析中药这一复杂体系并建立有效的质量控制方法，高效色谱分离技术发挥着关键作用，其中高效色谱分离材料的开发是技术核心。针对中药色谱分离中存在的问题，当前色谱材料的发展主要围绕4个关键方向：1）改善峰形，中药中的活性成分生物碱在分离中往往存在拖尾问题，从而影响分离度，因此要针对生物碱等峰形拖尾的化合物开发出专用固定相材料；2）提升分离选择性，比如针对皂苷异构体难分离的问题开发出具有正交性和选择性的固定相材料；3）加快分析速度，开发出亚2 μm的超高效色谱分离材料；4）扩大化合物覆盖范围，研制出具有更多普适性的多功能固定相材料。

基于上述发展脉络，为应对中药复杂体系分离分析中的挑战，人们致力于开发新型高效色谱分离材料。表1汇总了自2020年以来报道的新型高效色谱分离材料。下面将按照色谱分离模式分别进行阐述，包括反相液相色谱（RPLC）模式、亲水作用液相色谱（HILIC）模式、超临界流体色谱（SFC）模式这3类。

**表1:** 高效色谱分离材料汇总

Material	Abbr.	Mode	Analytes	Ref.
Fluorinated C18 bonded silica	FC8	RPLC	alkaloids	［^8^］
9-Oxa-10-phosphaphenanthrene-10-oxide-functionalized silica gel	DOPO-SiO_2_	RPLC	flavonoids	［^9^］
Phenyl vinyl sulfone stationary phase bonded silica	M-PVS	RPLC	saponins， flavonoids	［^10^］
Phenyl/amide/carboxyl bonded silica	PAC	RPLC	flavonoids， saponins， alkaloids， terpenoids	［^11^］
Cardanol-bonded on silica	CBS	RPLC	alkaloids	［^12^］
Hydrogenated rosin （*β*-acryloxyl ethyl） ester-bonded silica	HRE@SiO_2_	RPLC	paclitaxel	［^13^］
Propylene pimaric acid （16-hydroxyethyl acrylate-34-dodecyl） ester bonded silica	BRLA@SiO_2_	RPLC	saponins	［^14^］
3-Amino-5-mercapto-1，2，4-triazole bonded silica	AMTA	HILIC	flavonoids， saponins， alkaloids	［^15^］
Amide modified core shell silica	CS_X-E_-AM	HILIC	oligosaccharides	［^16^］
Ayrazine-2，3-dicarboxylic anhydride bonded silica	ZAC	HILIC	oligosaccharides， flavonoids， alkaloids， organic acids	［^17^］
*β*-Cyclodextrin stationary phase	*β*-CD	HILIC	steroids， saccharides	［^18^］
（1-Acrylamide propyl sulfonic） *_n_* -ImprSiO_2_	（AMPS） *_n_* -ImprSiO_2_	HILIC	flavonoids， fructus ligustri lucidi	［^19^］
Polyvinylpyrrolidone bonded silica	PVP@SiO_2_	HILIC	flavonoids， alkaloids	［^20^］
Sil-epoxy-polyethyleneimine	Sil-epoxy-PEI	HILIC	saccharides	［^21^］
Sil-chloropropyl-polyethyleneimine	Sil-chloropropyl-PEI	HILIC	saccharides	［^21^］
Methacryloxypropyl-trimethoxysilane -divinylbenzene-cysteine	MAPS-DVB-Cys	HILIC	alkaloids	［^22^］
*N*-Propylbenzamide	PB	SFC	flavonoids	［^23^］
4-Fluoro-*N*-propylbenzamide	PB-F	SFC	flavonoids	［^23^］
4-Ethyl-*N*-propylbenzamide	PB-ET	SFC	flavonoids	［^23^］
Aniline bonded silica	*S*-aniline	SFC	flavonoids	［^24^］
1-Aminonaphthalene bonded silica	*S*-1-ami-naph	SFC	flavonoids	［^24^］
1-Aminoanthracene bonded silica	*S*-1-ami-anth	SFC	flavonoids	［^24^］
1-Aminopyrene bonded silica	*S*-1-ami-py	SFC	flavonoids	［^24^］
L-Cysteine bonded silica	Click TE-Cys	SFC	tanshinones	［^25^］
3-Amino-5-mercapto-1，2，4-triazole bonded silica	AMTA	SFC	alkaloids	［^26^］
C8 bonded strong anion exchange silica	C8SAX	SFC	alkaloids	［^27^］
C8 bonded strong cation exchange silica	C8SCX	SFC	alkaloids	［^27^］
4-（Allylamino）butane-1-sulfonic acid	ZEX1	SFC	basic analytes	［^28^］
4-（Prop-2-yn-1-ylamino）butane-1-sulfonic acid	ZEX2	SFC	basic analytes	［^28^］
3-（Allylamino）propane-1-sulfonic acid	ZEX3	SFC	basic analytes	［^28^］
4-（Allyloxy）butane-1-sulfonic acid	SCX1	SFC	basic analytes	［^28^］
4-（Pent-4-en-1-yloxy）butane-1-sulfonic acid	SCX2	SFC	basic analytes	［^28^］
4-（Undec-10-en-1-yloxy）butane-1-sulfonic acid	SCX3	SFC	basic analytes	［^28^］
Ordered mesoporous silica	OMS	SFC	tanshinones	［^29^］
Ordered mesoporous core-shell silica	OMCS	SFC	Glycosides	［^30^］

### 1.1 反相色谱分离材料

RPLC是最常用的色谱分离模式，其分离机制主要基于疏水固定相与溶质分子之间的疏水相互作用^［31，32］^。该模式具有优异的样品覆盖度，能够适用于大部分中等极性到弱极性化合物及复杂样品基质的高效分离^［33］^。反相模式的一个突出优势在于其拥有成熟的色谱柱供应体系，包括沃特世科技（上海）有限公司、安捷伦科技有限公司、岛津公司和月旭科技（上海）股份有限公司等知名色谱技术企业均可提供多样化的反相固定相产品。许多反相固定相凭借其独特的选择性、高效的分离效率和广泛的分析物覆盖范围，在中药活性成分如生物碱、黄酮、皂苷和萜类等的分离分析和制备纯化领域发挥着重要作用^［34］^。然而，传统反相色谱固定相存在一个明显的技术局限：对极性化合物的保留能力和选择性不足^［35］^。随着化合物种类和分离要求的日益多样化，新型反相色谱分离材料仍在不断发展中。当前的研究重点在于保持材料疏水性的同时，通过结构设计和功能优化，进一步提升其选择性和分离性能，以满足更复杂的分离需求。

在RPLC领域，十八烷基键合硅胶（C18）固定相是最为广泛使用的反相色谱分离材料，其次为辛烷基键合硅胶（C8）短链固定相，苯基和芳基键合固定相通常作为C18和C8固定相的补充选择^［31］^。近年来，通过结构修饰提升固定相性能成为研究热点。Song等^［8］^在C8固定相的基础上开发了氟原子修饰的全氟辛基硅胶（FC8）固定相，该材料对碱性化合物展现出更强的保留和更优的分离选择性。在苯基固定相的创新设计方面，Xiao等^［9］^开发了一种新型苯基固定相——9-氧杂-10-磷杂菲-10-氧化物修饰硅胶（DOPO-SiO_2_）。该材料通过苯环和含磷基团的协同作用，对非极性至中等极性芳香族化合物均表现出优异的分离能力，拓展了固定相的极性适用范围。为进一步提升分离性能，Xiao等^［10］^开发了基于苯基乙烯基砜（M-PVS）的多功能键合配体固定相，其对具有相似疏水骨架但不同极性取代基的化合物展现出优异的分离选择性，这主要源于材料表面硫醚和砜基团与溶质分子之间形成的特定分子相互作用。特别值得关注的是，Zhao等^［11］^通过在苯基固定相中引入酰胺和羧基等极性基团，成功制备了邻苯二甲酸酐二氧化硅基（PAC）新型反相固定相。该材料不仅展现出与C18不同的分离选择性，还具有很好的正交性，成为构建RPLC×RPLC二维分离系统的固定相选择。当前反相色谱分离材料的发展致力于在传统C18/C8/苯基/芳基的基础上引入氟原子、磷、硫醚、酰胺和羧基等极性基团进行功能化修饰，这不仅显著提升了材料对特定分析物的分离选择性和保留能力，还成功构建出具有正交性的新型材料，为中药等复杂样品的分离分析提供了新的技术方案。

在新型RPLC固定相的开发中，也常利用天然产物作为功能化配体，这主要源于其具有来源广泛和官能团丰富等优势^［12，14，36］^。Zeng等^［12］^以天然产物腰果酚为配体，成功制备了腰果酚键合硅胶固定相（CBS）。该材料不仅保留了疏水作用，还兼具*π-π*相互作用和氢键作用，对烷基苯、多环芳烃和苯酚类化合物展现出良好的分离选择性。在天然产物固定相研究中，松香是一种天然二萜类树脂酸，具有双键、羧基和类菲稠环骨架，类似于常规C18功能配体。Li等^［13］^开发了一种由氢化松香（*β*-丙烯酰氧基乙基）酯键合硅胶（HRE@SiO_2_）组成的化学键合固定相，表现出典型的反相色谱行为和优异的色谱性能。Tanaka测试表明HRE@SiO_2_柱的疏水性不如C18柱，但其形状选择性更佳。考虑到松香衍生物可能是化学键合硅基有前途的功能配体，Xie等^［14］^进一步以丙烯海松酸（16-羟乙基丙烯酸-34-十二烷基）酯（BRLA）为功能配体开发了新型BRLA@SiO_2_反相色谱分离材料。松香独特的三元菲结构增强了固定相的空间选择性，且空间选择性随着碳链长度的增加而增加。BRLA@SiO_2_中羧基的存在增强了对极性溶质的氢键作用，导致其对极性溶质具有更好的分离选择性。以腰果酚、松香等天然产物作为功能配体，可赋予RPLC固定相丰富的多重分子间作用力，从而形成有别于传统C18固定相的独特分离选择性，显著改善了对多类化合物的分离性能。

### 1.2 亲水色谱分离材料

HILIC作为RPLC的重要补充技术，在强极性和可电离化合物的分离中具有独特优势^［21］^。该技术最早由Alpert于1990年首次提出，其分离机制主要是基于分析物分子在HILIC固定相表面的富水层和流动相中有机溶剂之间的多重相互作用，包括亲水液液分配、氢键和静电相互作用^［37，38］^。相较于传统RPLC技术，HILIC不仅增强了强极性和亲水性化合物的保留，而且还提供了互补的分离选择性^［39］^。这一技术优势使其在中药复杂体系研究中展现出重要价值，已成功实现糖苷类、寡糖类、甾体类及酚酸类等极性化合物的高效分离和制备纯化^［34，40］^。为了进一步扩展HILIC在中药分析中的应用，开发具有多种相互作用基团和大量亲水作用位点的新型HILIC固定相逐渐成为当前的研究热点。

HILIC常用的亲水极性固定相主要分为两类：一类是正相液相色谱的传统固定相，如裸硅胶、氨基、氰基和二醇；另一类是为HILIC设计的专用固定相，如酰胺、多元醇羟基和两性离子固定相^［15］^。尽管HILIC固定相种类丰富，但其在选择性和保留能力等方面仍存在改进空间。近年来，一系列新型HILIC固定相被相继报道。例如，Sheng等^［18］^开发了一种新型*β*-环糊精（*β*-CD）修饰的硅胶色谱固定相，用于分离蟾蜍提取物中的蟾蜍二烯内酯。该固定相通过疏水空腔包裹甾体部分，同时其表面羟基与精氨酸部分发生强亲水相互作用。此外，*β*-CD对多种糖类化合物（如核糖和山梨糖）也表现出优异的分离性能，其洗脱顺序符合亲水色谱极性规律。Zhao等^［16］^开发了一种具有可调厚度与孔径的核壳微球，经酰胺改性后制得亲水型核壳（CS_X-E_-AM）固定相。与传统的酰胺改性全多孔硅胶柱相比，CS_X-E_-AM在分离不同聚合度的巴戟天寡糖时展现出更优异的分离度和更短的分析时间，证明亲水核壳固定相在实现高效快速分离方面的显著优势。Jin等^［17］^成功研制了一种基于硅胶基质的（ZAC）亲水固定相，该材料采用2，3-吡嗪二羧酸酐和氨基作为键合基团，展示出优异的色谱分离性能。通过引入含氮杂环、酰胺及羧基等多功能配体，使固定相能同时提供亲水、*π-π*、氢键和静电相互作用等多种分离机制。基于含氮杂环配体提供的多种相互作用，Song等^［15］^进一步开发了以3-氨基-5-巯基-1，2，4-三唑为配体的硅胶（AMTA）亲水固定相。该材料通过氮杂环共轭体系实现了电荷分布的精准调控，并结合羟基和硫醚的协同作用增强了极性选择性。AMTA材料展现出以下突出特点：优异的通用性、广泛的选择性、良好的峰形、独特的分离选择性、增强的保留能力以及更高的稳定性。多种亲水化合物在AMTA柱上均具有良好的保留和峰形，可作为商品化HILIC柱和C18柱的补充。新型HILIC分离材料通过设计*β*-环糊精、基于核壳结构的酰胺以及含氮杂环等多功能配体，有效克服了传统材料在选择性与保留能力上的不足，在对复杂极性样品的分离中展现出卓越的通用性、选择性和分析效率。

与低相对分子质量改性硅胶固定相相比，聚合物改性硅胶固定相能够形成更厚的富水层，从而显著增强对极性化合物的亲水保留能力。Shu等^［19］^以球形硅胶为基质，通过逐步接枝咪唑、酰胺及磺酸基团，成功构建了一种新型表面聚合亲水固定相（（AMPS） *_n_* -ImprSiO_2_）。该材料表面功能基团丰富，兼具优异亲水性与离子交换特性，显著提升了分离选择性。Chen等^［20］^开发的聚乙烯吡咯烷酮键合硅胶（PVP@SiO_2_）固定相，凭借对多羟基结构的选择性识别能力，在酚类化合物分离中表现出卓越性能。Zhao等^［21］^通过将聚乙烯亚胺（PEI）接枝到硅胶表面，合成了两种专门用于糖类分析的Sil-epoxy-PEI和Sil-chlorpropyl-PEI亲水固定相。聚合物改性硅胶固定相通过表面接枝多功能聚合物层，有效提升了亲水保留能力与分离选择性，在极性化合物分离中展现出显著优势。

相较于硅胶基质固定相，聚合物固定相具有更宽的pH耐受范围。Yang等^［22］^制备了5 µm单分散有机硅烷杂化聚合物微球，该材料展现出优异的机械强度和低溶胀特性，经半胱氨酸修饰后得到MAPS-DVB-Cys亲水色谱固定相。与商品化半胱氨酸修饰硅胶柱相比，该固定相对酸碱化合物展现出更优的分离选择性，并在高pH流动相条件下表现出更好的耐碱性。开发具有高强度和低溶胀特性的聚合物基质固定相，是拓展HILIC在极端pH条件下应用的有效策略。

### 1.3 超临界流体色谱材料

SFC是一种采用超过临界温度和临界压力的流体作为流动相的色谱分析技术^［41］^。最常用的超临界流体是二氧化碳（CO_2_），其极性接近己烷或戊烷等弱极性溶剂^［42］^。与高效液相色谱（HPLC）所用的液体流动相相比，超临界CO_2_具有低黏度、高扩散系数和低传质阻力等特性^［43］^，因此SFC在多个方面展现出显著优势，例如可在高流速下维持较低系统压力、实现更短时间的高通量分析、获得更高的分离效率以及具备环境友好特性等^［44］^。

SFC不仅在手性分离领域得到广泛应用，展现出对手性异构体优异的分离选择性，其在非手性分离中的应用范围也在不断拓展。然而SFC在非手性分离中仍面临问题：其一，SFC主要集中在弱极性至中等极性化合物的分离上，而对于强极性化合物如生物碱的分离存在峰形拖尾、保留不够、和分离选择性差等问题^［45］^；其二，SFC所使用的固定相依然沿用HPLC的固定相，针对超临界流体特性开发的SFC专属固定相较少。因此，发展新的SFC固定相有助于扩大SFC的应用范围，解决中药中极性化合物的分离问题。

SFC的常用固定相包括2-乙基吡啶（2-ethylpyridine，2-EP）、2-吡啶胺（2-picolylamine，2-PIC）、二醇、二乙胺（diethyl amine，DEA）、1-氨基蒽（1-aminoanthracene，1-AA）、NH_2_和Amide等。近年来，Jiang等^［23］^合成了一系列带有不同键合基团的苯基固定相，包括*N*-丙基苯甲酰胺（PB）、4-氟-*N*-丙基苯甲酰胺（PB-F）和4-乙基-*N*-丙基苯甲酰胺（PB-ET）。通过在苯环上引入吸电子基团（氟）或给电子基团（乙基）来调节各种相互作用，从而扩展了在SFC中的应用。此外，Ge等^［24］^还制备了苯胺（*S*-aniline）、1-氨基萘（*S*-1-ami-naph）、1-氨基蒽（*S*-1-ami-anth）和1-氨基芘（*S*-1-ami-py）等一系列苯基固定相。该类固定相通过芳香基团提供*π-π*相互作用，嵌入的极性基团（2-羟基-3-丙氧基）丙胺增强氢键作用，从而有效确保极性溶质的保留和分离性能。Xia等^［25］^将半胱氨酸键合两性离子亲水固定相（Click TE-Cys）应用在SFC的分析上，实现了丹参酮的SFC高效分离分析。在脂溶性丹参酮类样品的分析中，相比于常见的SFC固定相和反相C18固定相，高极性的Click TE-Cys材料表现出更强的保留行为和更好的分离选择性。考虑到亲水固定相在SFC中的应用潜力，Song等^［26］^进一步将自制AMTA亲水固定相应用在SFC上，评估了其在碱性化合物分离中的表现。结果显示，相较于传统SFC固定相，AMTA可以显著改善碱性化合物的峰形，表明含氮杂环结构对于碱性化合物分离至关重要，因为它不仅提供电荷排斥效应，还有助于实现均匀的电荷分布。新型SFC固定相通过对苯环等骨架进行功能基团修饰（如引入氟、乙基），或直接采用两性离子、含氮杂环等亲水设计，有效调控了*π-π*、氢键及静电等多种相互作用，从而显著拓展了其分离选择性，并成功改善了碱性化合物等难分离物质的峰形。

近年来，混合模式固定相开始逐渐应用在SFC的分离分析上。例如Fu等^［27］^开发了基于两种反相/离子交换混合模式固定相（C8SAX和C8SCX）的SFC分析方法。这两种材料具有色散和静电相互作用，适用于碱性化合物的分离。在C8SAX固定相中，添加酸性缓冲盐可有效调节碱性化合物的保留行为，不仅延长其保留时间，同时显著提高了分离选择性。而在C8SCX固定相中，通过引入中性缓冲盐来优化碱性化合物的分离效果。Wolrab等^［28］^制备了6种混合模式固定相（zwitterion ion-exchange，ZEX1、ZEX2、ZEX3、SCX1、SCX2和SCX3），其结构中均含有可作为离子交换功能基团的磺酸基。通过调控烷烃链长度来调整固定相的疏水性，并在烃链中嵌入氧、氮等杂原子，引入额外的相互作用位点。在SFC模式下评估了这6种固定相对碱性分析物的分离性能，结果表明SCX2是使用不同流动相条件分离碱性分析物的最佳选择。在SFC中应用混合模式固定相，通过整合反相疏水作用与离子交换作用，并精准调控其结构，有效优化了对碱性化合物的保留行为、选择性与峰形。

在固定相种类不断发展的过程中，SFC固定相的粒径也在逐渐向更小的方向发展。Song等^［29］^制备了粒径为1.1 µm的有序介孔全多孔硅胶（OMS），并将其用作SFC固定相。凭借其单分散性好、孔道有序以及超临界流体促进传质的特性，OMS在SFC上实现了34万/m的超高柱效，可以在40 s内分离5种丹参成分。在此基础上，Feng等^［30］^进一步开发了1.2 µm有序介孔核壳硅胶（OMCS），该材料具有高度均一的颗粒单分散性和均匀的有序孔分布，在SFC下达到了32万/m的超高柱效。与1.7 µm的Waters BEH C18柱相比，1.2 µm的OMCS柱在SFC下表现出更好的分离选择性和更快的分离速度，并具有很好的正交性。目前新型SFC分离材料正向亚微米级的小粒径发展，以此实现超高柱效与快速分离，为中药等复杂体系的高效分析开辟了新途径。

## 2 高效分析色谱

高效分析色谱法旨在采用高效色谱分离材料快速分离中药中的复杂混合物，并与检测器在线联用，从而实现对中药中多种化学成分的高通量识别和快速结构解析。近年来，随着固定相技术的不断发展，多种新型高效色谱分离材料被应用于中药活性成分的分离分析。生物碱、黄酮、皂苷和萜类是中药中非常重要且研究较多的活性成分，下面将围绕这4类应用对象来分别进行阐述。

### 2.1 生物碱

生物碱是从植物中提取的具有生物活性的含氮有机物，具有宣肺平喘、镇痛、抗菌消炎和抗肿瘤等多种药理药效作用^［46］^。然而，生物碱的特殊结构也为其色谱分离带来了挑战，其中峰形拖尾和选择性差是常见的分离难题^［27］^。这主要源于生物碱分子中的氮原子与固定相表面残留硅醇基之间的相互作用，该效应不仅延长了保留时间，也减缓了吸附动力学速率，从而导致色谱峰形不对称，进而影响分离选择性^［47］^。

近年来，开发新型固定相以改善生物碱分析中常见的峰形拖尾和选择性不足等问题，已成为色谱研究的热点。Zeng等^［12］^将CBS材料应用于吴茱萸提取物的分离分析，表现出良好的分离性能。其中含量最高的两个成分——吴茱萸碱和吴茱萸次碱实现了有效分离，分离度为5.43，且峰形对称尖锐，表明CBS材料对生物碱具有优异的色谱性能。Bai等^［48］^设计了一种星型羟丙基环糊精聚合物键合硅胶手性固定相（star hydroxypropyl cyclodextrin polymer chiral stationary phase，Sil-Star-HPCD CSP），可同时分离金鸡纳树皮中多种手性喹啉生物碱成分。该材料兼具高密度环糊精单元和羟丙基结构，增强了氢键作用能力，适用于分离生物碱复杂体系中结构相似但氢键位点不同的化合物。Fu等^［27］^将制备的C8SCX材料应用于吲哚生物碱的快速分离。在与商品化固定相1-AA、二醇、2-EP、2-PIC和DEA的对比中，只有C8SCX可以快速实现去氢钩藤碱、异去氢钩藤碱、钩藤碱和异钩藤碱的基线分离。Song等^［8］^将自主研发的新型FC8材料应用于黄连生物碱组分的分析，并与商品化C8和（pentafluorophenyl，PFP）材料进行对比。结果显示FC8材料对黄连生物碱表现出更强的保留能力和更优的分离选择性，这主要归因于其表面高密度氟原子可有效调控硅醇基团的电离行为，从而显著改善峰形与分离效果。基于FC8材料在生物碱分离方面的优异性能，Song等^［49］^将其进一步应用于麻黄生物碱的分离分析。通过对比FC8、反相C18和亲水XBridge Amide材料的分离效果，仅FC8能够实现麻黄中5种生物碱（去甲麻黄碱、去甲伪麻黄碱、麻黄碱、伪麻黄碱和甲基麻黄碱）的基线分离，且具有良好的定量分析适用性。此外，Song等^［26］^还评估了马钱子、延胡索和大叶钩藤等中药提取物在AMTA材料上的分离性能。这些提取物的主要活性成分是具有抗炎镇痛作用的生物碱。在低上样量下，两种提取物在AMTA上均获得了良好的峰形；在上样量较高时，峰形仍保持对称，表明该固定相可以大大提高生物碱的负载能力并获得对称的色谱峰。这一现象可以用多位点吸附理论来解释：AMTA表面正电荷和带正电的生物碱之间存在静电排斥作用，阻止生物碱进入深层高能吸附位点，从而促进其在外层低能位点均匀吸附，获得对称色谱峰。采用一维分离通常难以满足复杂样品中多组分分离的需求，因此研究人员构建了多维分离色谱体系。例如，Si等^［50］^结合C18柱与多糖基手性柱IG-3，解决了不同品牌烟草及卷烟烟气中4对生物碱对映体（*R*/*S*-尼古丁、*R*/*S*-可替宁、*R*/*S*-假木贼碱、*R*/*S*-新烟草碱）的分离问题。Zhao等^［11］^开发了在线综合二维系统，用于分析富含苄基异喹啉生物碱、脱氢表雄酮生物碱和环肽生物碱的酸枣仁提取物。该系统采用新型PAC柱和C18柱构建，正交度达83.3%，共鉴定出32种生物碱。更重要的是，Xu等^［51］^系统评估了一系列色谱分离材料对多种生物碱的分离性能，制定了适用于二维液相色谱方法开发的色谱柱选择指南。基于该指南，Xu等^［51］^开发了一种用于分离大叶乌药中生物碱的二维液相色谱方法，所获色谱峰形对称，正交性高达80.3%，验证了该指南的可行性与实用性。为攻克生物碱分析中的峰拖尾与选择性难题，研究已从依赖单一作用力的传统固定相，发展为利用氟原子、环糊精、两性离子等功能基团提供的多重相互作用，在实现优异峰形、高选择性分离及对映体拆分方面展现出卓越性能，并推动了从高效一维到高正交性多维分离方法的发展。

### 2.2 黄酮

黄酮类化合物是一类重要的酚类化合物，具有防治心脑血管疾病、高血压和冠心病等多种药理活性^［52］^。该类化合物结构复杂，常通过羟基化、甲基化或糖基化等修饰成多种衍生物^［20，53］^。目前，传统的C18固定相是分离黄酮类化合物最常用的色谱分离材料^［54］^。然而，由于黄酮类化合物结构多样，C18材料在分离选择性方面仍存在一定局限。开发能与多酚结构形成氢键的新型固定相，对提升黄酮类化合物的分离效率和选择性具有重要的研究价值。

近年来，研究者致力于开发新型固定相以提升黄酮类化合物的分离性能。Chen等^［20］^采用PVP@SiO_2_材料进行黄酮类化合物的分离研究。在以甲醇-水为流动相的体系中，基于线性溶剂化能量关系确定的系统常数表明，PVP@SiO_2_较C18可提供更强的氢键和*π-π*相互作用。在芹菜素、槲皮素、槲皮苷、木犀草素、鼠李素和山柰酚混合物的分离中，PVP@SiO₂表现出优于C18的分离选择性。等^［9］^比较了新型DOPO-SiO_2_固定相与商品苯基固定相对二氢黄酮类化合物的分离效果，两者均能有效分离儿茶素、二氢杨梅素、紫杉叶素、圣草酚和柚皮素的混合物。但DOPO-SiO_2_柱的分离时间显著缩短，更适合大批量黄酮样品的高通量分析。Jiang和Ge等^［23，24］^将所制备的7种新型苯基固定相（PB、PB-F、PB-ET、*S*-aniline、*S*-1-ami-naph、*S*-1-ami-anth和*S*-1-ami-py）应用于黄酮类化合物的SFC分离。当仅使用甲醇作为改性剂时，固定相与黄酮类化合物之间较强的氢键和*π-π*相互作用易导致色谱峰拖尾；而在甲醇中添加0.1%的磷酸后，显著改善了分离效果，获得了良好的色谱性能。Jin等^［17］^比较了不同HILIC柱对4种黄酮类化合物的分离效果，与Zwitterionic（ZIC）、Amide和PFP相比，分析物在ZAC柱上的峰形和保留效果非常好。其中化合物槲皮素-3-O-*β*-D-葡萄糖苷和槲皮素3-O-*β*-D-（6″-对-香豆酰）吡喃葡萄糖基（1-2）-*α*-L-鼠李吡喃糖苷在ZAC柱上的分离度最好。与上述一维液相色谱相比，多维色谱具有更高的选择性、分离度和峰容量。例如，Dai等^［55］^构建了一种以自制M-PVS柱（一维）与C18柱（二维）为核心的高正交性离线二维液相色谱系统，并将其应用于药用石首乌中的化学成分分析，成功鉴定出66种黄酮类化合物。Kuang等^［56］^则建立了基于Amide（一维）和Hypersil GOLD柱（二维）的HILIC×RPLC离线二维液相色谱-高分辨质谱联用方法，系统分析了补肾活血方中的化学成分，共鉴定出50种黄酮类化合物。为提升黄酮类化合物的分离效能，研究从新型固定相与多维系统两方面取得进展：多种功能化固定相通过增强氢键、*π-π*等相互作用，有效改善了选择性、峰形与分析效率；而基于不同分离机理构建的多维色谱系统，则大幅提升了复杂样品中黄酮类化合物的鉴定数量与分离能力。

### 2.3 皂苷

皂苷由亲水性聚糖与疏水性苷元连接而成^［57，58］^。常见的聚糖包括葡萄糖、木糖、阿拉伯糖、鼠李糖和半乳糖等^［59］^，常见的苷元包括三萜皂苷元和甾体皂苷元。皂苷具有多种重要的生物活性和药理作用，如抗肿瘤、抗炎、抗病毒和改善心血管等功能^［60］^。然而，构成聚糖部分的单糖残基极其多样化，很大程度上决定了皂苷的多样性和结构复杂性，这使其在色谱分离中面临巨大挑战^［61，62］^。例如，在常规C18色谱柱上，人参皂苷Rg1与Re往往难以实现基线分离，且洗脱时间较长^［63］^。因此，开发新型固定相以高效分离皂苷类化合物，具有重要的研究价值和应用前景。

近年来，研究人员陆续报道了多种新型色谱分离材料在皂苷类化合物分离中的应用。例如，Xie等^［14］^采用BRLA@SiO_2_材料对三七粗提取物中的皂苷类成分进行分离分析，实现了三七皂苷R1、人参皂苷Rg1、Re、Rb1及Rd的基线分离。该研究表明，BRLA@SiO_2_材料可替代传统的C18材料用于三七皂苷的高效分离。Xiao等^［10］^比较了11种皂苷和3种皂苷元在M-PVS柱、苯基柱和C18柱上的分离性能。仅M-PVS能有效分离人参皂苷Rh1和Rg2，这归因于该材料与糖基之间的额外极性相互作用。此外，分析物在苯基柱和C18柱上的保留均强于M-PVS柱，因其苯基和长烷基链疏水性更强，而M-PVS柱中引入的硫醚和砜基则降低了固定相的疏水性。Feng等^［30^还采用新开发的SFC专用材料OMCS，在6 min内实现了三七皂苷和人参皂苷实际样品的快速高效分离。除人参皂苷Rg1/Re难以分离外，Rb1/Rg2皂苷对在C18柱上也表现出较差的分离效果。通过比较11种皂苷混合物在OMCS和C18柱上的洗脱行为，发现两者保留特性存在显著差异，并展现出良好的正交性，表明OMCS柱在皂苷类样品分离中具有较好的互补分离潜力。多维色谱联用技术已成为解决复杂中药体系中皂苷类成分分离分析的重要工具。Dai等^［55］^开发了一套高正交性离线二维液相色谱系统，该系统以自制的M-PVS柱（一维）和C18柱（二维）为核心，并将其用于分析药用石首乌中的化学成分，成功鉴定出64种皂苷类化合物。Zhao等^［11］^则开发了基于一维自制PAC柱与二维C18柱的在线二维分离系统。该PAC固定相疏水性低于C18材料，在在线系统中表现出良好的溶剂相容性。研究人员将这一系统用于分析富含达玛烷型三萜皂苷的酸枣仁提取物，成功鉴定出30种皂苷类成分。针对皂苷类化合物，新型固定相通过引入氢键、极性等互补作用力，在选择性、分析速度上超越了传统C18。基于此构建的多维色谱系统，则凭借其高正交性，构成了高效分离与鉴定复杂中药皂苷类成分的关键技术工具。

### 2.4 萜类

萜类化合物是由两个或多个异戊二烯单元聚合而成的一类天然产物^［64］^。根据分子骨架中所含异戊二烯单元的数目，萜类可分为单萜、倍半萜、二萜、三萜、四萜和多萜等^［65］^。除了基本的烃类结构外，萜类更常以醇、醛、羧酸和糖苷等含氧衍生物的形式存在^［66］^。该类化合物具有抗菌、抗炎和抗肿瘤等多药理活性，但其结构的复杂性和性质的多样性也对色谱分离技术提出了更高要求^［59］^。因此，开发能够实现高效和高选择性分离的新型材料，已成为萜类化合物深入研究与应用的关键。

近年来，随着多种新型分离材料的不断涌现和发展，萜类化合物的分离分析取得了显著进展。以生育酚为例，其侧链为典型的二萜结构单元，其中*β*和*γ-*生育酚因结构极其相似，传统C18材料难以分离这一对异构体。针对该问题，Malik等^［67］^以异氰酸十八酯为原料，通过酯化和苄基脱保护反应，制备了*β*-丙氨酸衍生的尿素和酰胺的C18固定相（*β*-alanine-derived C18 stationary phase containing embedded urea and amide groups，Sil-Ala-C18），然后将其键合于（3-氨丙基）三甲氧基硅烷修饰的二氧化硅上。Sil-Ala-C18材料结合了酰胺基和脲基的协同作用，可同时提供氢键作用、亲水性与润湿性调控以及羰基-*π*相互作用，从而在*β*与*γ-*生育酚异构体的分离中表现出优异的分离选择性。丹参酮作为中药丹参中的主要脂溶性成分，属于具有特征性呋喃D环的醌型松香烷二萜类化合物。由于其结构复杂多样性，开发有效的色谱分离材料对深入研究丹参酮类成分的化学组成与生物活性至关重要。然而传统C18材料对丹参酮类成分表现出极弱的保留，几乎无法实现有效分离。针对这一挑战，Xia等^［25］^制备的Click TE-Cys材料成功实现了11种丹参酮类成分（包括丹参新醌B、丹参新酮、丹参酮ⅡA、甘西鼠尾新酮A、丹参酮甲酯、丹参酮Ⅰ、丹参醇A、隐丹参酮、二氢丹参酮Ⅰ、新隐丹参酮和丹参新醌A）的高效分离分析。与C18和常规极性色谱柱相比，高极性的Click TE-Cys显著增强了丹参酮类化合物的保留行为与分离选择性。为了提高丹参酮的分离效率并缩短分析时间，Song等^［29］^进一步制备了小粒径的超高效分离材料OMS，并将其应用于丹参酮提取物的快速分离。与商品化PIC、氨基、二醇和裸硅胶柱相比，OMS能够在40 s内实现对5种主要丹参酮成分（丹参酮ⅡA、丹参酮Ⅰ、隐丹参酮、四氢丹参酮Ⅰ和二氢丹参酮Ⅰ）的高效快速分离。相比之下，商品柱不仅洗脱时间更长，分离度也较差。这一优异性能归因于OMS材料优异的单分散性和有序的孔道结构，从而赋予其超高的柱效。新型分离材料通过氢键作用、极性调控及孔道结构优化，显著提升了萜类化合物的分离选择性与分析效率，突破了传统C18材料的局限性。

## 3 高效制备色谱

化合物的纯化是中药单体化合物获取和活性研究的必要步骤，借助制备色谱技术，可高效地从复杂的中药混合物中大规模分离并获取高纯度目标化合物^［68］^。传统制备色谱通常采用直径较大的玻璃或不锈钢柱管，依靠空气泵或重力驱动流动相流动，具有设备简单、成本较低的优点，适用于实验室常规纯化需求^［69］^。然而，该方法也存在分离效率低、重复性差、分离度有限以及自动化程度低等局限^［70］^。高效制备色谱是一种通过高上样量、高分辨率的制备柱实现高纯度分离的液相色谱技术。与传统制备色谱相比，高效制备色谱具有更高的分离效率、更高的自动化程度、可重复和可工程化等优势^［71］^，已逐步成为本草物质组研究中的核心技术。

近年来，多种新型色谱填料被开发并应用于中药化合物的制备纯化，显著提升了目标成分的纯度。研究者针对不同类型的中药成分设计了一系列功能化固定相。如在不饱和脂肪酸的纯化过程中，以C18为固定相的RPLC是常用方法。Jin等^［72］^制备了低（C18AD）、中（C18ME）、高（C18HD）3种键合密度的C18材料，通过比较乙基棕榈油酸的峰展宽和浓度分布，发现C18ME材料展现出最优的色谱性能。进一步采用该填料对原油混合物进行两步纯化，乙基棕榈油酸含量从34.58%提升至96.57%，符合高纯度脂肪酸产品的要求。在黄酮类成分的纯化中，Chen等^［20］^开发的PVP@SiO_2_兼具硅胶的机械强度和聚合物在宽pH范围内的稳定性，相比常规C18材料，其更强的氢键和*π-π*相互作用使其对黄酮类化合物表现出优异的分离能力，已成功应用于银杏黄酮的制备纯化。在甜菊糖苷的纯化方面，Wang等^［73］^开发的聚丙烯酰胺基键合硅胶填料（polyacrylamide，PA）表现出优异的亲水性和高选择性，分离性能优于商品化二醇、酰胺和氨基柱等HILIC固定相。将PA柱用于醇/水体系中低丰度糖苷的纯化，成功获得两种高极性糖苷，纯度均超过98%。在紫杉醇的制备纯化中，Li等^［13］^基于紫杉醇与松香结构的相似性，合成了松香键合硅胶HRE@SiO_2_材料，实现了紫杉醇的高效纯化，纯度从6%提升至82%。在生物碱纯化方面，氟化反相固定相（FC8HL）表现出优于C18的选择性。Si等^［74］^采用氟化反相固定相FC8HL实现了贝母中甾体生物碱的高效富集。该固定相在高比例有机相中对碱性化合物具有较高的保留性能。通过在制备型FC8HL色谱柱上进行生物碱的大规模富集，最终从260 g贝母原料中获得186 mg的甾体生物碱。各类新型色谱填料凭借其特异性相互作用和优异的分离性能，为中药复杂体系中不同类别活性成分的高效制备纯化提供了技术支撑。

在填料技术不断创新的基础上，多维制备色谱技术进一步整合多种分离材料，在中药活性成分的高效制备纯化中展现出应用潜力^［75-79］^。如Dai等^［77］^通过将直链淀粉三（3，5-二甲基苯基氨基甲酸酯）和纤维素三（4-甲基苯甲酸酯）分别键合至硅胶载体，构建了基于两种手性固定相的制备型二维SFC系统，实现了海风藤中非手性脂肪酸衍生物的高效纯化。Dai等^［78］^采用二维RPLC×HILIC策略，成功从白花蛇舌草中分离纯化出高极性环烯醚萜苷和黄酮化合物。该方法第一维采用制备型C18柱进行初步纯化，第二维则采用制备型苯基柱，利用其*π-π*相互作用有效弥补了C18固定相在选择性上的局限，最终共纯化出27个化合物，主要涉及9个环烯醚萜苷和5个黄酮类成分。为应对更复杂的生物碱体系，Liu等^［76］^开发了一种离线三维制备型HPLC方法，用于钩吻属植物中生物碱的制备纯化。该方法第一维采用C18柱，第二维为C8CE柱，第三维为C18CE柱，各维固定相之间均表现出良好的正交性。最终成功分出24个吲哚生物碱，其中包括5个新化合物。该团队^［79］^进一步建立了基于多维、多模式、多柱（3M）策略的生物碱高效纯化方法。该方法第一维借助离子交换柱HCSX实现生物碱的初步富集；第二维和第三维分别采用反相柱C18和C18CE进行正交分离；第四维使用氟化反相固定相FC8HL分离结构高度相似的组分；第五维则通过反相C18柱在高分离度条件下完成高纯度化合物的最终纯化。通过3M制备色谱策略，从13.7 kg防己中成功纯化出81种生物碱，其中包括37个新化合物和7种具有新颖骨架结构的生物碱。多维制备色谱技术通过整合多种分离材料，实现了对中药复杂体系中活性成分的高效纯化与深度解析，显著提升了化合物纯化的效率与新结构的发现能力。

## 4 总结与展望

近年来，本草物质组研究在色谱分离方法学、结构解析及生物活性研究等方面取得了显著进展，其中新型高效色谱分离材料发挥了重要作用。多种色谱模式材料如反相、亲水和SFC等材料不断涌现，显著提升了中药复杂体系中生物碱、黄酮、皂苷和萜类化合物的分离选择性和分析速度。随着高效色谱分离材料种类的增多，其在本草物质组研究中的应用也越来越具有针对性。研究人员在方法开发初期即可根据目标化合物进行色谱柱选型，避免大规模盲目筛选色谱柱，显著提升了分离方法开发的效率与准确性。此外，这些新型高效色谱分离材料也在制备色谱领域实现了突破：一方面通过改善峰形和选择性，提升了分离效率；另一方面使得以往难以获取的微量活性成分得以高效纯化，极大地丰富了可分离的化合物数量，为本草物质组研究提供了丰富的物质基础。

尽管本草物质组研究已取得显著进展，然而中药体系的复杂性仍是色谱分离技术需要面对的严峻挑战，并对色谱材料提出了更高要求。未来高效色谱分离材料的发展重点聚焦以下几个方向：一是提升分离选择性，开发具有正交分离特性的材料，增大色谱峰容量，从而提升对中药化合物的分析与制备能力；二是提升分离效率，开发粒径更小、机械强度更高的超高效色谱填料，推动耐压性能更好的超高效色谱仪器的发展，实现更快速的分离与更高的分离效率；三是发展通用型固定相，通过优化材料结构拓宽其适用范围，增强材料的普适性和选择性，以少数几种材料满足多类中药化合物的分离需求；四是提高制备色谱的分离能力，需要开发高载量、高稳定性的制备色谱材料，提高分离纯化的产额与通量。总之，未来高效色谱分离材料的发展应注重分离选择性与正交性、分离效率、通用性的统一，以支持本草物质组更高效、更系统化的化合物发掘与功能研究。

## References

[1] Liu S， Yi L Z， Liang Y Z . J Sep Sci， 2008， 31（11）： 2113 18615809 10.1002/jssc.200800134

[2] Lin A X H， Chan G， Hu Y J， et al . Chin Med， 2018， 13（1）： 9 29449877 10.1186/s13020-018-0167-z PMC5807832

[3] Zhou X， Li C G， Chang D， et al . Medicines， 2019， 6（1）： 14 30669335 10.3390/medicines6010014 PMC6473719

[4] Jia P， Bian Y Y， Bai Y J， et al . Chinese Journal of Chromatography， 2021， 39（9）： 950 34486834 10.3724/SP.J.1123.2021.06021 PMC9404139

[5] Liang X M . Research on the Herbalome// New Viewpoints and New Theories Academic Salon Series No. 23—Drug Discovery： Seeking Weapons to Safeguard Human Health. Beijing： China Science and Technology Press， 2009： 60

[6] Liang X M， Qian X H， Hui Y Z . World Science and Technology-Modernization of Traditional Chinese Medicine， 2007， 9（5）： 1

[7] Zhang X L， Liu Y F， Guo Z M， et al . Anal Bioanal Chem， 2012， 402（2）： 573 22089819 10.1007/s00216-011-5533-y

[8] Song C Y， Yu D P， Jin G W， et al . Chromatographia， 2022， 85（5）： 447

[9] Xiao Q， Guo D D， He J， et al . Anal Chim Acta， 2024， 1311： 342735 38816164 10.1016/j.aca.2024.342735

[10] Xiao R T， Shen A J， Jin G W， et al . J Chromatogr A， 2020， 1617： 460807 31889519 10.1016/j.chroma.2019.460807

[11] Zhao X W， Yu D P， Zhou W J， et al . J Sep Sci， 2023， 46（10）： 2200704 10.1002/jssc.202200704 36896497

[12] Zeng L， Jiang L J， Yao X D， et al . Chinese Journal of Chromatography， 2022， 40（6）： 547 35616200 10.3724/SP.J.1123.2021.12023 PMC9404126

[13] Li H， Xie W B， Zeng L， et al . J Chromatogr A， 2022， 1665： 462815 35038614 10.1016/j.chroma.2022.462815

[14] Xie W B， Li H， Zeng L， et al . Anal Chim Acta， 2023， 1239： 340661 36628701 10.1016/j.aca.2022.340661

[15] Song C Y， Jin G W， Guo Z M， et al . J Chromatogr A， 2024， 1734： 465315 39216280 10.1016/j.chroma.2024.465315

[16] Zhao X Y， Zhao C X， Wu R F， et al . J Chromatogr A， 2025， 1748： 465864 40088528 10.1016/j.chroma.2025.465864

[17] Jin G W， Ding J J， Zhou Y Z， et al . J Chromatogr A， 2021， 1638： 461825 33450715 10.1016/j.chroma.2020.461825

[18] Sheng Q Y， Wang L， Zhang L Y， et al . J Chromatogr A， 2022， 1673： 463069 35489243 10.1016/j.chroma.2022.463069

[19] Shu Y， Chen T， Liu T， et al . Microchem J， 2023， 191： 108863

[20] Chen X， Li J T， Gu M T， et al . Sep Sci Plus， 2023， 7（5）： 2300059

[21] Zhao X Y， Niu Y F， Zhao C X， et al . Int J Mol Sci， 2024， 25（17）： 9465 39273411 10.3390/ijms25179465 PMC11395661

[22] Yang Y， Li Y X， Zhao Y， et al . J Sep Sci， 2024， 47（17）： 2400462 10.1002/jssc.202400462 39252172

[23] Jiang D S， Ke Y X， Cai J F， et al . J Chromatogr A， 2020， 1614： 460700 31740031 10.1016/j.chroma.2019.460700

[24] Ge D D， Yang J， Yu Z M， et al . J Chromatogr A， 2024， 1716： 464640 38219626 10.1016/j.chroma.2024.464640

[25] Xia D H， Song C Y， Feng J， et al . J Chromatogr A， 2025： 466270 10.1016/j.chroma.2025.466270 40782475

[26] Song C Y， Jin G W， Yu D P， et al . J Chromatogr A， 2024， 1720： 464811 38490143 10.1016/j.chroma.2024.464811

[27] Fu Q， Dong W W， Ge D D， et al . J Chromatogr A， 2023， 1705： 464163 37348226 10.1016/j.chroma.2023.464163

[28] Wolrab D， Frühauf P， Kolderová N， et al . J Chromatogr A， 2021， 1635： 461751 33285414 10.1016/j.chroma.2020.461751

[29] Song C Y， Qi Y， Jin G W， et al . Chem Commun， 2024， 60（27）： 3649 10.1039/d3cc05690b 38372355

[30] Feng J， Song C Y， Xia D H， et al . J Chromatogr A， 2025， 1757： 466151 40540895 10.1016/j.chroma.2025.466151

[31] Žuvela P， Skoczylas M， Liu J J， et al . Chem Rev， 2019， 119（6）： 3674 30604951 10.1021/acs.chemrev.8b00246

[32] Dong X F， Cai X M， Shen A J， et al . Chinese Journal of Chromatography， 2013， 31（4）： 297 23898625 10.3724/sp.j.1123.2012.12009

[33] Zhou W J， Liu Y M， Wang J X， et al . J Sep Sci， 2020， 43（1）： 87 31664759 10.1002/jssc.201900765

[34] Jin H L， Liu Y F， Guo Z M， et al . J Pharm Biomed Anal， 2016， 130： 336 27329167 10.1016/j.jpba.2016.06.008

[35] Guo Z M， Zhang X L， Xu Q， et al . Chinese Journal of Chromatography， 2009， 27（5）： 675 20073204

[36] Zeng L， Cao Y， Yao X D， et al . Chinese Journal of Chromatography， 2020， 38（11）： 1257 34213095 10.3724/SP.J.1123.2020.07039

[37] Lardeux H， D'Atri V， Guillarme D . TrAC-Trends Anal Chem， 2024， 176： 117758

[38] Xu G G， Yi Y， Liu P P， et al . Chinese Journal of Chromatography， 2025， 43（7）： 734 40610768 10.3724/SP.J.1123.2025.04015 PMC12231493

[39] Cavazzini A， Catani M， Felinger A . Liq Chromatogr， 2023： 227

[40] Buszewski B， Noga S . Anal Bioanal Chem， 2012， 402（1）： 231 21879300 10.1007/s00216-011-5308-5 PMC3249561

[41] Broeckhoven K . TrAC-Trends Anal Chem， 2022， 146： 116489

[42] Chen M， Wen S S， Wang R， et al . Molecules， 2022， 27（13）： 4159 35807405

[43] Si-Hung L， Bamba T . TrAC-Trends Anal Chem， 2022， 149： 116550

[44] Sun T T， Luo J Y， Xu Y Y， et al . J Pharm Biomed Anal， 2021， 199： 114039 33839642 10.1016/j.jpba.2021.114039

[45] Firooz S K， Wahab M F， Yu J， et al . Talanta， 2021， 232： 122308 34074384 10.1016/j.talanta.2021.122308

[46] Rajput A， Sharma R， Bharti R . Mater Today Proc， 2022， 48： 1407

[47] Cass Q B， Tiritan M E， Junior J M B， et al . Chiral Separations and Stereochemical Elucidation： Fundamentals， Methods， and Applications. Ontario： JohnWiley & Sons， 2023

[48] Bai H， Zhao Y N， Chen L . Talanta， 2025， 295： 128369 40408999 10.1016/j.talanta.2025.128369

[49] Song C Y， Yu D P， Jin G W， et al . J Sep Sci， 2022， 45（5）： 1051 34984820 10.1002/jssc.202100645

[50] Si X X， Tian Y， Xu S F， et al . Rev Anal Chem， 2024， 43（1）： 20230074

[51] Xu Y， Liu Y F， Zhou H， et al . Talanta， 2023， 251： 123738 35921743 10.1016/j.talanta.2022.123738

[52] Mutha R E， Tatiya A U， Surana S J . Futur J Pharm Sci， 2021， 7（1）： 25 33495733 10.1186/s43094-020-00161-8 PMC7816146

[53] Janas P， Bocian S， Jandera P， et al . J Chromatogr A， 2016， 1429： 198 26709027 10.1016/j.chroma.2015.12.024

[54] Lesellier E， West C . J Sep Sci， 2022， 45（1）： 382 34633729 10.1002/jssc.202100567

[55] Dai Y B， Zhang K L， Xiong L， et al . J Sep Sci， 2022， 45（10）： 1727 35297180 10.1002/jssc.202200013

[56] Kuang W， Wang Y， Huang Y X， et al . J Sep Sci， 2024， 47（2）： 2300624 10.1002/jssc.202300624 38286726

[57] Wang Y， Ma Y， Tao L， et al . Separations， 2022， 9（7）： 163

[58] Savarino P， Demeyer M， Decroo C， et al . Mass Spectrom Rev， 2023， 42（3）： 954 34431118 10.1002/mas.21728

[59] Song C Y， Jin G W， Yu D P， et al . Chinese Journal of Chromatography， 2023， 41（10）： 866 37875409 10.3724/SP.J.1123.2023.07024 PMC10599299

[60] Xu X H， Li T， Fong C M V， et al . Molecules， 2016， 21（10）： 1326 27782048 10.3390/molecules21101326 PMC6272920

[61] Nguyen H T， Vu-Huynh K L， Nguyen H M， et al . Molecules， 2021， 26（17）： 5373 34500805 10.3390/molecules26175373 PMC8433671

[62] Huang Y， Zhang T T， Zhou H B， et al . J Pharm Biomed Anal， 2016， 121： 22 26773536 10.1016/j.jpba.2015.12.056

[63] Xie W B， Li H， Sun Y， et al . Microchem J， 2022， 176： 107234

[64] Huang W Q， Wang Y X， Tian W S， et al . Antibiotics， 11（10）： 1380

[65] Feng M X， Li C Y， Wang C， et al . Int J Food Prop， 2022， 25（1）： 2445

[66] Ninkuu V， Zhang L， Yan J P， et al . Int J Mol Sci， 2021， 22（11）： 5710 34071919 10.3390/ijms22115710 PMC8199371

[67] Malik A K， Montero L， Meckelmann A W， et al . Anal Chem， 2023， 95（14）： 6172 37005395 10.1021/acs.analchem.3c00697

[68] Niculau E S， Oliveira D A B， Almeida N F . Sep Sci Plus， 2022， 5（11）： 602

[69] Kondeti R R， Mulpuri K S， Meruga B . World J Pharm Sci， 2014： 1375

[70] Zhao S S， Wang C Y， Wang X， et al . J Chromatogr A， 2020， 1619： 460917 32037073 10.1016/j.chroma.2020.460917

[71] Xie X M， Sun W Y， Huang J Y， et al . Chin J Anal Chem， 2016， 44（7）： 1140

[72] Jin G W， Liu L J， Yang Y L， et al . J Sep Sci， 2022， 45（11）： 1866 35324071 10.1002/jssc.202101021

[73] Wang J， Zhao Y， Yang Y， et al . J Food Compos Anal， 2022， 112： 104683

[74] Si W， Yu D P， Zhou H， et al . J Sep Sci， 2021， 44（18）： 3441 34291571 10.1002/jssc.202100379

[75] Zhu J B， Li W Y， Xiao W， et al . Chinese Journal of Chromatography， 2018， 36（5）： 464 30136488 10.3724/SP.J.1123.2017.12025

[76] Liu D， Han Y， Zhou H， et al . J Chromatogr A， 2021， 1640： 461935 33556681 10.1016/j.chroma.2021.461935

[77] Dai Z S， Jiang D S， Dai Y P， et al . J Chromatogr B， 2022， 1188： 123079 10.1016/j.jchromb.2021.123079 34906822

[78] Dai Y P， Zhang H Z， Wang X H， et al . J Sep Sci， 2023， 46（10）： 2300029 10.1002/jssc.202300029 36880199

[79] Liu D， Han Y， Chen Y N， et al . Talanta， 2025， 293： 128054 40203600 10.1016/j.talanta.2025.128054

